# *In vitro* investigation of metabolic fate of α-mangostin and gartanin via skin permeation by LC-MS/MS and in silico evaluation of the metabolites by ADMET predictor™

**DOI:** 10.1186/s12906-020-03144-7

**Published:** 2020-11-23

**Authors:** P. Rukthong, N. Sereesongsang, T. Kulsirirat, N. Boonnak, K. Sathirakul

**Affiliations:** 1 Department of Pharmacy, Faculty of Pharmacy, Mahidol University, Bangkok, Thailand; 2 Department of Chemistry, Faculty of Science, Thaksin University, Songkhla, Phatthalung Thailand

**Keywords:** Skin permeation, Skin metabolism, *Garcinia mangostana* L.;α-Mangostin, Gartanin, ADMET predictor

## Abstract

**Background:**

Mangosteen, Garciniam angostana L., is a juicy fruit commonly found in Thailand. The rinds of Garciniam angostana L.have been used as a traditional medicine for the treatment of trauma, diarrhea and skin infection. It is also used in dermatological product such as in cosmetics. The mangosteen pericarp can be used to extract valuable bioactive xanthone compounds such as α-mangostin and gartanin. This study is aimed to predict the metabolism of α-mangostin and gartanin using in silico and in vitro skin permeation strategies.

**Methods:**

Based on their 2D molecular structures, metabolites of those compounds were predicted in silico using ADMET Predictor™. The K_m_ and V_max,_ for 5 important recombinant CYP isozymes 1A2, 2C9, 2C19, 2D6 and 3A4 were predicted. Moreover, the in vitro investigation of metabolites produced during skin permeation using human epidermal keratinocyte cells, neonatal (HEKn cells) was performed by LC-MS/MS.

**Results:**

It was found that the results derived from in silico were in excellent alignment with those obtained from in vitro studies for both compounds. The prediction referred that gartanin and α-mangostin were the substrate of CYP1A2, 2C9, 2C19 and 3A. In the investigation of α-mangostin metabolites by LC-MS/MS system, the MW of the parent compound was increased from 411.200 to 459.185 Da. Therefore, α-mangostin might be metabolized via tri-oxidation process**.** The increased molecular weight of parent compound (397.200 to 477.157 Da) illustrated that gartanin might be conjugated to sulfated derivatives.

**Conclusions:**

In all the studies, α-mangostin and gartanin were predicted to be.

metabolized via phase I and phase II metabolism (sulfation), respectively.

## Background

Mangosteen (*Garcinia mangostana* L.) is a popular tropical fruit that grows mainly in Southeast Asia and other tropical areas. In Thailand, it has been used as a traditional medicine for the treatment of trauma, diarrhea and skin infections [1]. It is used in dermatological products such as in cosmetics. Mangosteen contains two major bioactive xanthone compounds, α-and γ-mangostins, that are major bioactive compounds. The biological activities of xanthone compounds are antimicrobial activity against methicillin-resistant *Staphylococcus aureus* [2], inhibition of oxidative damage by human low-density lipoprotein (LDL) [3] and weak antioxidant activity [4]. Skin is a major interface between the environment and the body. Previous studies revealed that P-gp has a physiologic function during the migration of dendritic cells from skin via lymphatic vessels [5] and that keratinocytes show a high expression of CYP enzymes, such as CYP2B19 [6]. Uptake of xenobiotics into normal human epidermal keratinocytes (HEKn) and subsequent excretion of these substances has long been thought to be a process of passive diffusion. Other studies by Abels et al. [7] showed that the cellular uptake of indocyanine green in HaCaT keratinocytes is inhibited by bromosulfophthalein indicating the possible involvement of an organic anion transporting polypeptide (OATP) in the active uptake of organic cations like indocyanine green. Also, normal human epidermal keratinocytes have been shown to express a cell-type-specific pattern of extrahepatic cytochrome P450 enzymes and efflux transport proteins showing that these cells metabolize and excrete a variety of xenobiotics [8]. Cultured skin alternatives have appeared during the last decade, and there is an increasing demand for good cell culture models that can be used in pharmaceutical and toxicological studies. Cultured skin models can remain viable for long period in vitro experiments, as long as culture conditions are maintained. This allows more opportunities to perform more elaborate experiments [9]. In silico research, in which mathematical models of a physiologic or pharmacologic system are developed and tested on a computer, are a hybrid of in vivo and in vitro techniques. This techniques, thus, offer the clinician-scientist the opportunity to answer questions that, for a variety of reasons, could not otherwise be easily addressed [10]. Many conferences have included sessions that focus on the in silico prediction of ADME properties and toxicity, highlighting the interest in moving these approaches to a point at which they can be used routinely in the drug-discovery process [11–13]. The application of in silico modelling and simulation within drug development is rapidly increasing in the R&D sector of the pharmaceutical industry [14]. The in silico approach can be used from ADMET property predication to clinical trial simulation. It was used to predict the drug-like properties of Chinese herbal compounds include the virtual screening method, pharmacophore model method and machine learning method [15]. For a metabolism prediction, in silico tools are most commonly used for predicting substrates and inhibitors of metabolic enzymes, sites of metabolism, and the structures of probable metabolites. ADMET predictor™ was used to predict of CYP isoforms potentially involved in phytocannabinoid metabolism. The software correctly predicted several of the many metabolites reported in the literature for the main phytocannabinoids [16].

Accordingly, the purpose of this study was to predict the metabolite of α-mangostin and gartanin for skin permeation and characterize the metabolism of α-mangostin and gartanin in human epidermal keratinocyte cells, neonatal (HEKn cells). The concentrations of the xanthone compounds were determined by LC-MS/MS and the metabolite of two xanthones compounds were predicted by in silico method using ADMET Predictor™ program.

## Method

### Plant material

The *G. mangostana* is a common tropical fruit consumed by household in Thailand. The hull of *G. mangostana* was collected in June 2011 at Chumporn Province, Southern part of Thailand. Botanical identification was achieved through comparison with a voucher specimen of Assoc. Prof. Dr. Pichaet Wiriyachitra, which was deposited in the herbarium of Department of Biology, Prince of Songkla University.

**Extraction and Isolations.**

**Fig. 1:**
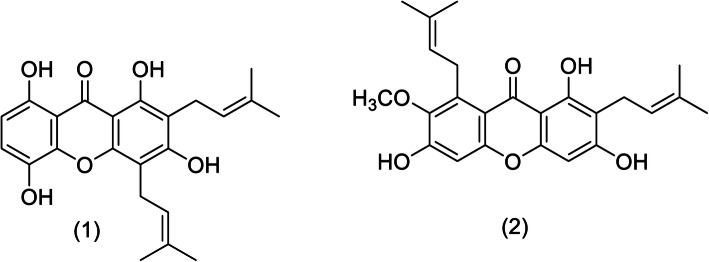
The Chemical Structure of compounds 1 and 2

The crude CH_2_Cl_2_ extract (260 mg) of the hulls of *G. mangostana* was subjected to quick column chromatography (QCC) on silica gel (Merck 60 F_254_) using hexane as a first eluent and then increasing the polarity with Acetone to give 21 fractions (**F1**-**F21**). Fraction **F3** was further separated by column chromatography (CC) eluting with a gradient of EtOAc-hexane to give 39 subfractions (**F31**-**F339**). Subfraction **F35** was purified by LH20 eluting with 25% H_2_O-MeOH to afford 9 subfractions (**F35A**-**F35I**) and gartanin (5.2 mg). Fraction **F8** was further separated by CC eluting with a gradient of EtOAc-hexane to give 16 subfractions (**F81**-**F816**) and α-mangostin (336.3 mg) (Fig. 1) [17, 18].

**Compound (1)**: Gartanin*.* Yellow powder, m.p. 168–170 °C; ^1^H NMR 300 MHz (CDCl_3_) *δ* (ppm): 12.29 (1H, *s*, 1-OH), 11.20 (1H, *s*, 8-OH), 7.17 (1H, *d*, *J* = 9.0 Hz, H-6), 6.60 (1H, *d*, *J* = 9.0 Hz, H-7), 6.54 (1H, *s*, 3-OH), 5.18 (1H, *m*, H-2′), 3.40 (2H, *d*, *J* = 6.9 Hz, H-1′), 1.72 (1H, *s*, H-4′), 1.51 (1H, *s*, H-5′), 5.20 (1H, *m*, H-2′′), 3.46 (2H, *d*, *J* = 6.9 Hz, H-1′′), 1.79 (1H, *s*, H-4′′), 1.69 (1H, *s*, H-5′′).

**Compound (2)**: *α*-mangostin*.* Deep-yellow powder, m.p. 180–182 °C; UV-Vis (CHCl_3_) λ_max_ (log *ε*) 243 (3.90), 289 (4.10), 298 (4.13), 320 (3.68), 351 (3.47), 391 (3.33) nm; FT-IR (neat) ν_max_ 3397, 1649, 1613 cm^− 1^. ^1^H NMR 300 MHz (CDCl_3_) *δ* (ppm): 13.77 (1H, *s*, 1-OH), 6.29 (1H, *s*, H-4), 6.82 (1H, *s*, H-5), 3.45 (2H, *d*, *J* = 7.2 Hz, H-1′), 5.29 (1H, *m*, H-2′), 1.77 (1H, *s*, H-4′), 1.84 (1H, *s*, H-5′), 4.09 (2H, *d*, *J* = 6.0 Hz, H-1′′), 5.26 (1H, *m*, H-2′′), 1.69 (3H, *s*, H-4′′), 1.84 (3H, *s*, H-5′′), 3.81 (3H, *s*, 7-OCH_3_).

### In vitro study

#### Primary cell culture

**Fig. 2:**
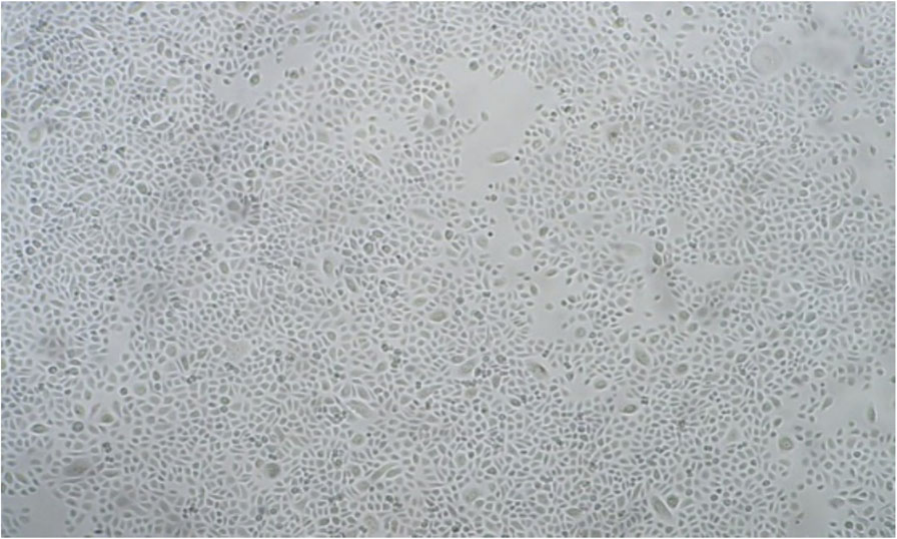
Schematic representation of the HEKn cells monolayer grown on a permeable support. The dotted line represents the surfaces of the experimental medium in the two chambers

Human epidermal keratinocyte (neonatal) (HEKn) (InvitrogenTM, Cat# C-001-5C Lot#1654391, Oregon, USA) cells were grown in T-25 flask at 37 °C in an atmosphere of 5% CO_2_. The adherent keratinocytes were cultured in low calcium (0.09 mM), serum-free, Epilife® medium, and supplement with Human Keratinocyte Growth Supplement (HKGS) at 1% v/v concentration. When a 500 ml of Epilife® medium was supplemented with HKGS the final concentration of the components in the supplement medium were: bovine pituitary extract (BPE) 0.2% v/v, bovine insulin 5 μg/ml, hydrocortisone 0.18 μg/ml, bovine transferrin 5 μg/ml and human epidermal growth factor 0.2 ng/ml. The medium was replaced regularly three times a week until the flask reaches 90% confluence (Fig. 2). The cells were moved from the flasks by incubating the monolayers with 0.5% trypsin for 2–3 min at 37 °C. The cells were collected into centrifuge tubes, and then centrifuged at 1000 rpm for 4 min and the pellets were resuspended in Epilife® medium. Cells used for this study were in third and fourth passage in late sub confluency.

#### Culturing of HEKn cells on permeable supports

**Fig. 3:**
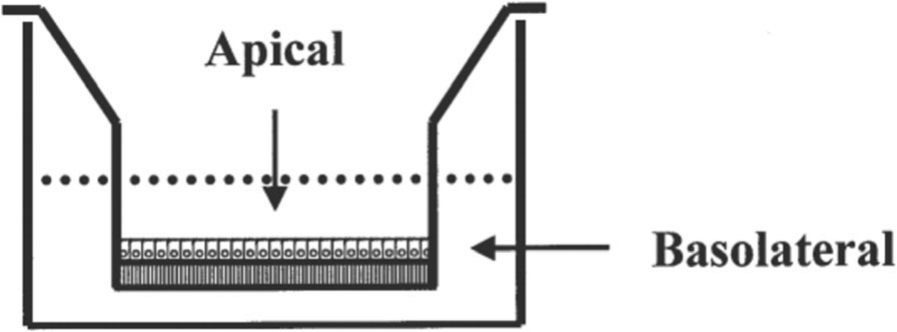
HEKn cells were grown in T-25 flask at 37 °C in an atmosphere of 5% CO_2_ and reach 90% confluence (10X)

HEKns cells were seeded at a density of 1 × 10^5^ cells/cm^2^ on permeable supports (Transwell® Costar, 0.4 μm pore size) as shown in Fig. 3. The complete media were hanged every other day. Before the experiments, the cells were washed with D-PBS. Then, the xanthone compounds dissolved in DMSO were added into the cell cultures. DMSO (0.1%) treatment was used as control. The integrity of the HEKns cell monolayer in the transwell plates was determined by measuring Trans Epithelial Electrical Resistance (TEER). Acceptable TEER value is > 400 Ω •.cm^2^ by using Millicell ERS-2 Voltohmmeter (Sigma- Aldrich, Germany). Moreover, the mannitol was used as a marker compound for paracellular transport permeation. The cells were incubated at 37 °C at eight time points (i.e. on days 1,3, 5, 7, 10, 14, 20, and 21).

### In silico study (in silico prediction)

ADMET Predictor™ version 8, formerly known as QMPRPlus™, a state-of-the-art computer program, was used to estimate certain ADMET properties of gartanin and α-mangostin from their molecular structures. The program required 2D or 3D molecular structure information, parses the structure and calculates the values of molecular descriptors. The program uses the molecular descriptor values as inputs to independent mathematical models (generally, nonlinear machine learning techniques) in order to generate estimates for each of the ADMET properties. It provided a human CYP450 enzyme kinetic parameters including *K*_*m*_ and *V*_*max*_ models for five important recombinant CYP isozymes which were 1A2, 2C9, 2C19, 2D6, and 3A4 and qualitative assessment of the molecules as human CYP450 substrate and inhibition was predicted.

### Liquid chromatography-mass spectrometry

#### LC-MS/MS analysis

The LC-MS/MS system consisted of a Q-Trap 5500 triple quadrupole/ion trap mass spectrometer (ABSCIEX™, USA) equipped with a Turbo Spray ion source operated at 350 °C. The pMRM–IDA-EPI (Predicted Multiple reaction monitoring mode – Information dependent acquisition - Enhanced production scan) method used pMRM (predicted MRM) as a survey scan. The information-dependent acquisition (IDA) method was employed to trigger the enhanced product ion (EPI) scans by analyzing MRM signals. A total of 66 MRM transitions were used as the survey experiment in positive mode. The system included an Agilent 1200 HPLC pump, degasser, auto sampler and column heater (Agilent, USA). The in house developed and validated HPLC separation was performed using a 50 × 2.1 mm, Luna 5 μm NH_2_ 100 Å column (Agilent, USA) operated at a flow rate of 1000 μl/min. A mobile phase system consisted of (A) water and (B) acetonitrile and was used with the following gradient: 30% B for 0.75 min, 30 to 90% B from 0.75 to 7 min, and finally 30% B isocratic from 7 to 12 min.

## Results

### Metabolism of gartanin and α-mangostin

**Fig. 4:**
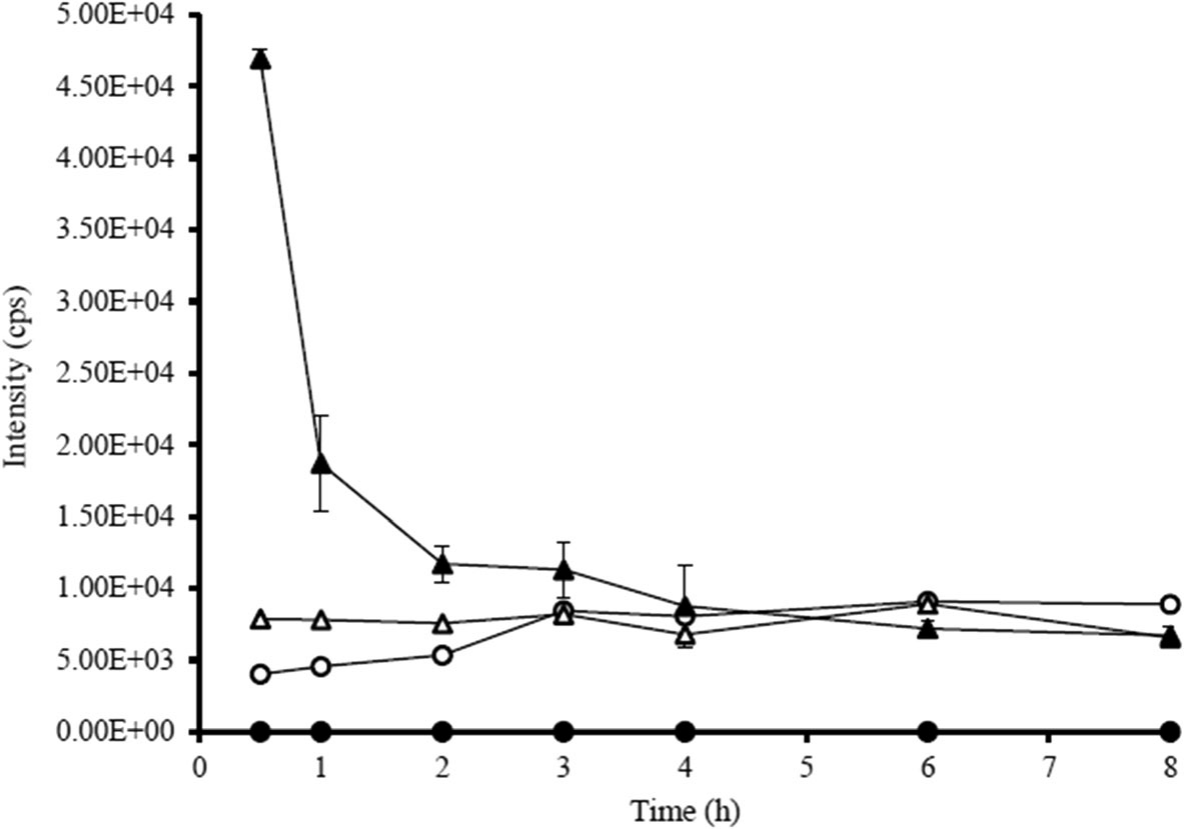
The intensity (cps) of the parent and conjugated gartanin in absorptive and secretory directions. ( , gartanin in absorptive direction; , conjugated gartanin in absorptive direction; , gartanin in secretory direction; , conjugated gartanin in secretory direction)

**Fig. 5:**
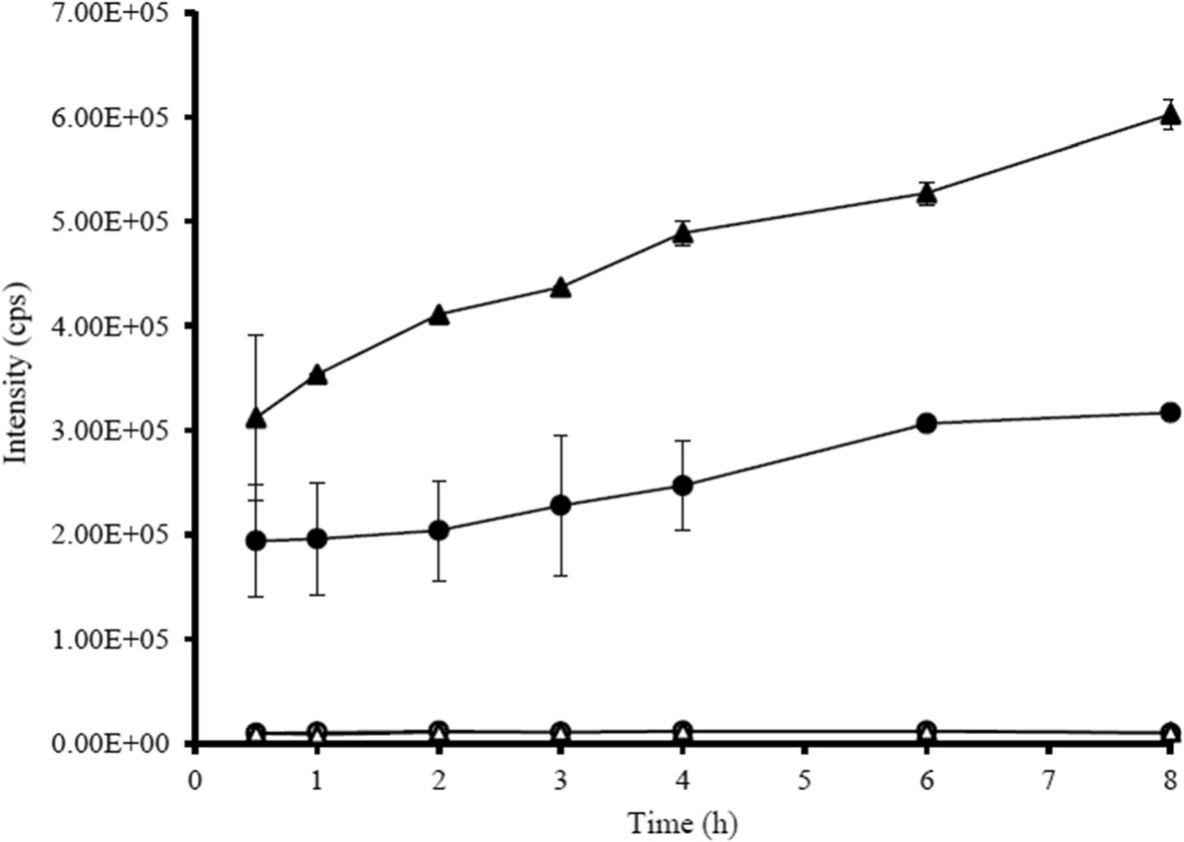
The intensity (cps) of the parent and oxidized α-mangostin in absorptive and secretory directions. ( , α-mangostin in absorptive direction; , oxidized α-mangostin in absorptive direction; , α-mangostin in secretory direction; , oxidized α-mangostin in secretory direction)

**Fig. 6:**
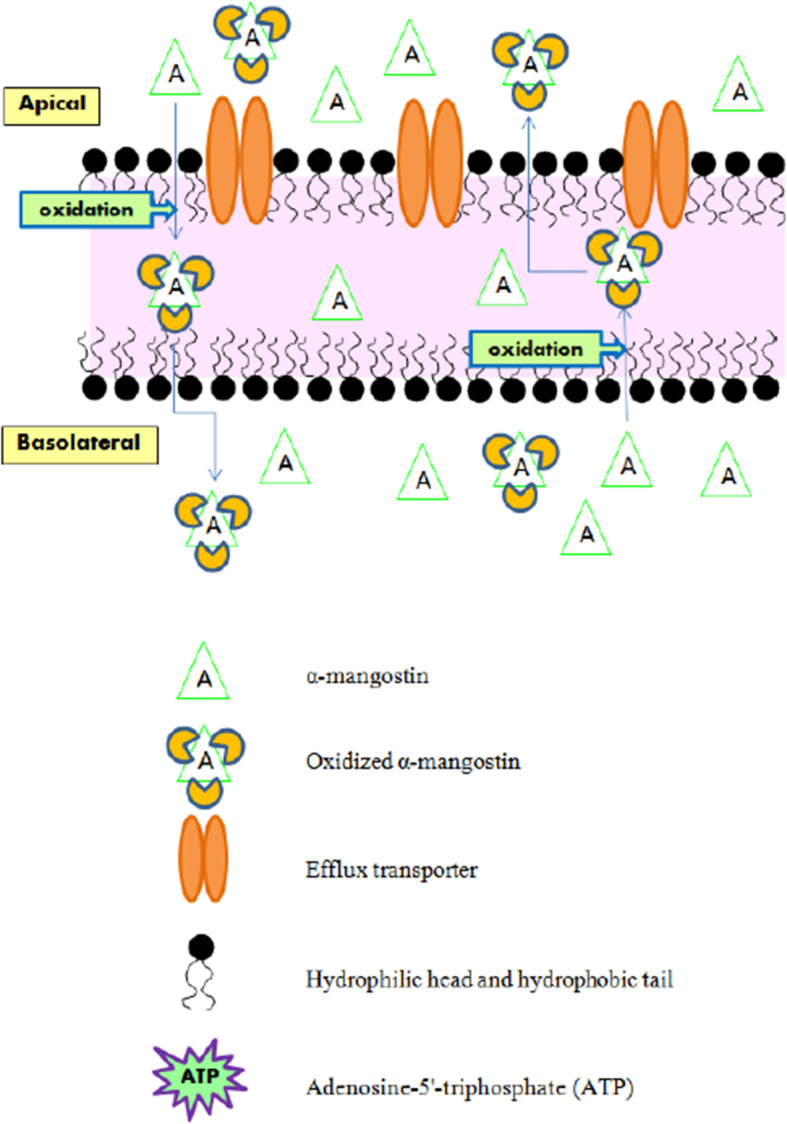
The transport of gartanin and conjugated gartanin across the HEKn cells in both absorptive and secretory directions

**Fig. 7:**
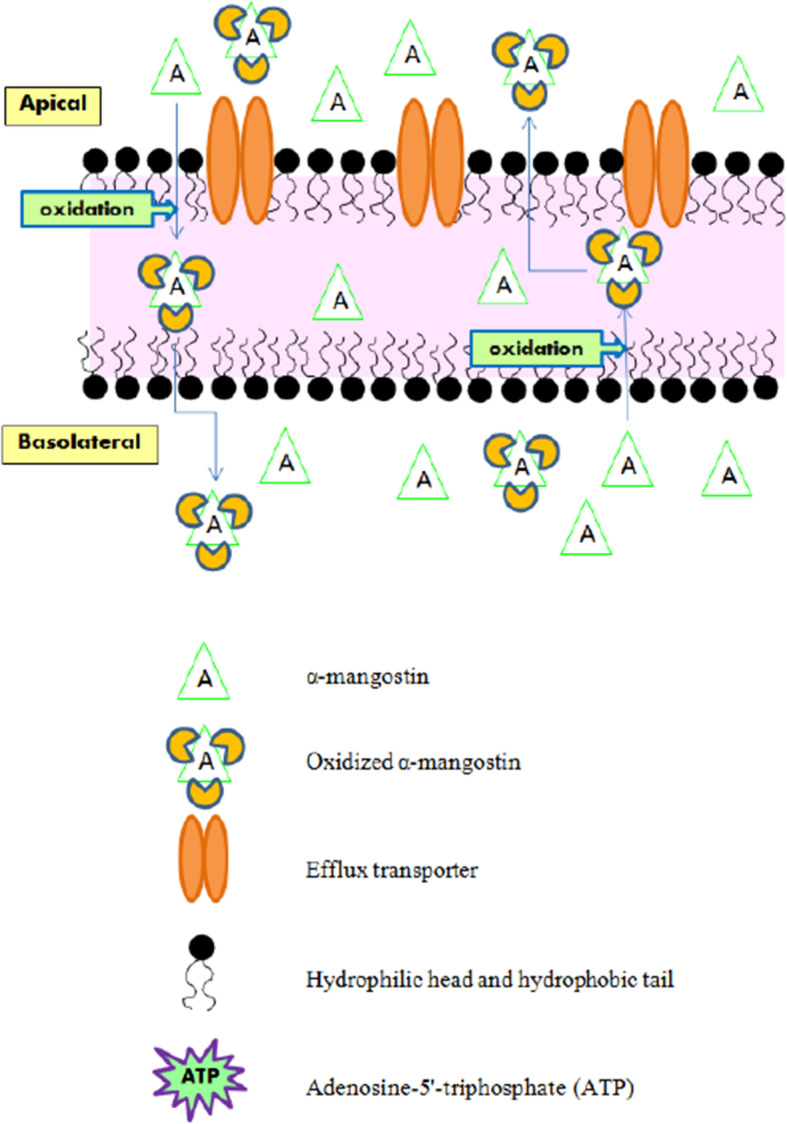
The transport of α-mangostin and oxidized α-mangostin across the HEKn cells in both absorptive and secretory directions

Two xanthones with different substitutions were investigated on a LC-MS/MS system. Total 10 processes were run in positive scan mode. There were oxidation (+ 15.995 Da), di-oxidation (+ 31.990 Da), tri-oxidation (+ 47.985 Da), hydrogenation (+ 2.061 Da), sulfation (+ 79.957 Da), methylation (+ 14.016 Da), di-methylation (+ 28.032 Da), glucuronidation (+ 176.034 Da), de-methylation (− 14.016 Da) and de-hydrogenation (− 2.016 Da). Common features were displayed that gartanin and α-mangostin exhibited gain of small neutral molecules from the precursors. These were helpful to summarize the mechanism of two xanthone compounds. The increased molecular weight (MW) of parent compounds (397.200 to 477.157 Da) illustrated that gartanin might be conjugated to sulfated derivatives. The conjugated gartanin could permeate from apical to basolateral side and vice versa as showed in Fig. 4 and there were almost stable at the intensity approximately 8.00E+ 3 cps. This indicated that the conjugated gartanin could not have the ability to attach the efflux transporters as the parent compounds. Thus, the metabolite accumulated in both apical and basolateral side of the cells. For α-mangostin, the MW of parent compound was increased from 411.200 to 459.185 Da. Therefore, α-mangostin might be metabolized via tri-oxidation process. In addition, the intensity was increased within 30 min then constant in both absorptive and secretory directions and the intensity were equal in apical and basolateral side (Fig. 5). The transport of gartanin and α-mangostin in both parent compounds and metabolites were illustrated in Fig. 6-7.

### In silico prediction by ADMET predictor™

The program obtained 2D molecular structure information and then parsed the structure and calculated the values of molecular descriptors.

### Simulation of apparent permeability coefficient (P_app_)

The simulations plus S + MDCK model predicted P_app_ measured using the MDCK (Madin-Darby Canine Kidney) cells-on-sheet (COS) technology. The calculation informed that P_app_ of gartanin and α-mangostin were 52.62 and 93.51 nm/sec, respectively. When P_app_ was compared to the P_app_ from the previous experiments [16] P_app, A-B_ of gartanin (9 μM) could not be detected and P_app, A-B_ of α-mangostin was lower than predicted 3-fold. These could be concluded that these two compounds might be permeated through the intestinal better than skin when using each compound alone.

### In silico prediction of drug metabolism

**Fig. 8:**
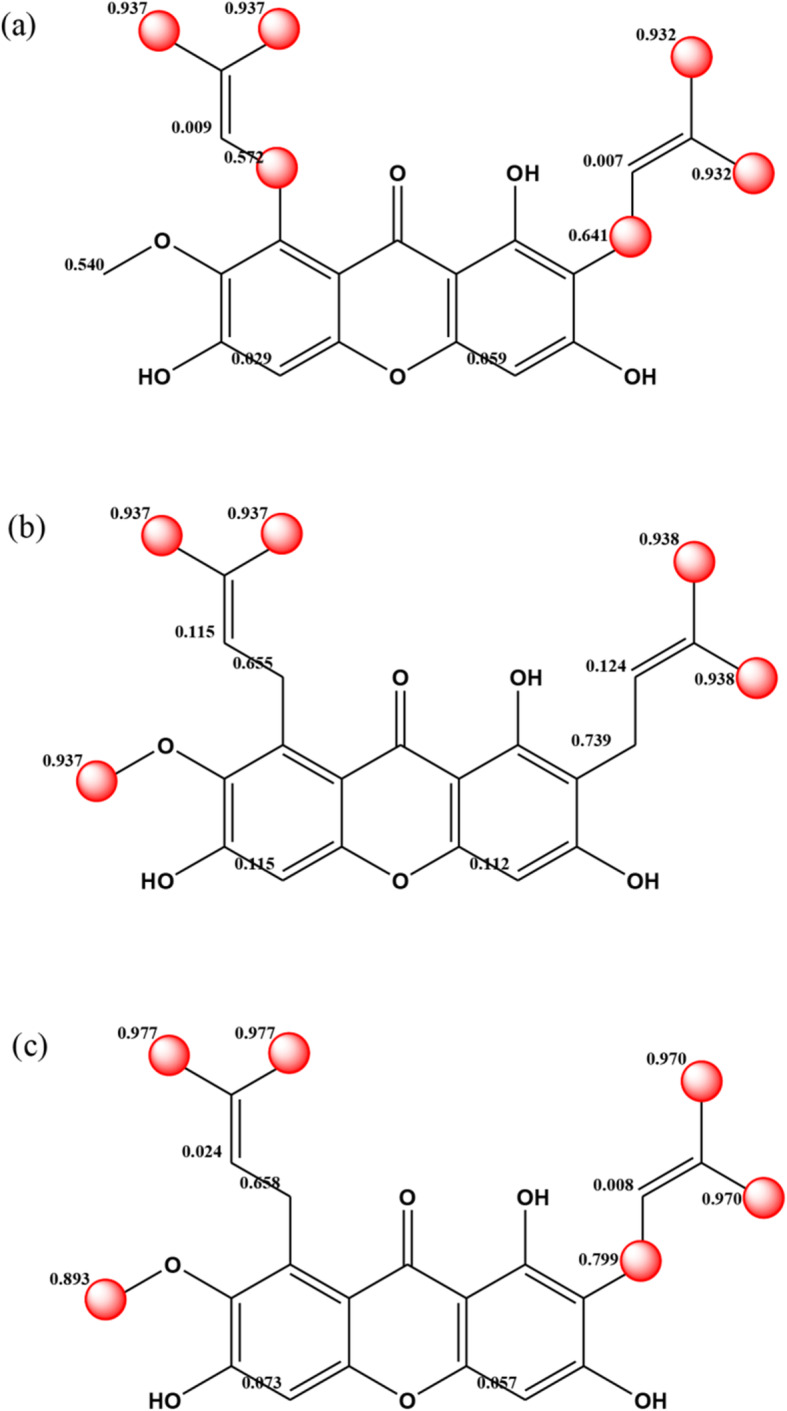
The possible atomic site(s) of α-mangostin by CYP 1A2, 2C9 and 2C19 metabolism

**Fig. 9:**
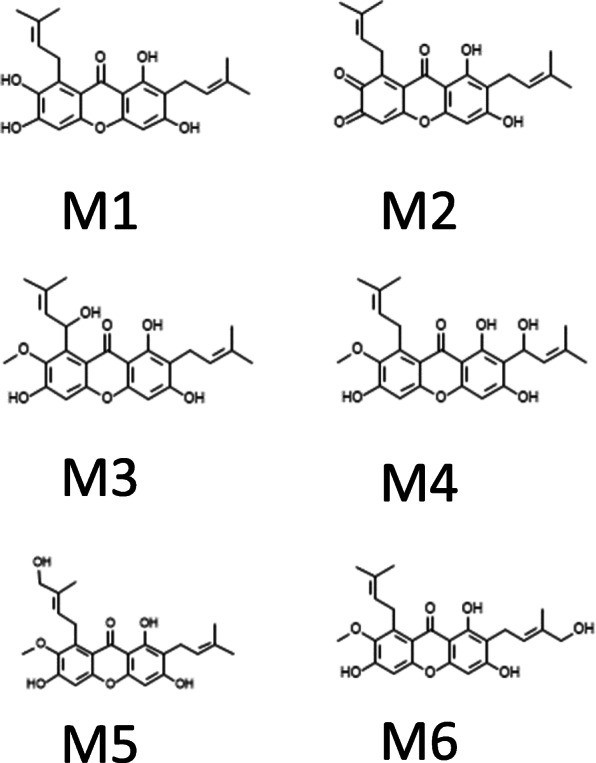
The possible phase I oxidation of α-mangostin (M1-M6)

ADMET Predictor™ provided a human CYP450 enzyme kinetic models including *K*_*m*_ and *V*_*max*_ models for five important recombinant CYP isozymes which were 1A2, 2C9, 2C19, 2D6, and 3A4. By using the 2D molecular structure of gartanin and α-mangostin, qualitative assessment of a molecule being the human CYP450 substrate and inhibition were shown in Table 1. The gartanin and α-mangostin were predicted to be a substrate of CYP1A2, 2C9, 2C19 and 3A4. The intrinsic clearance for each CYP isozymes of both molecules were also estimated by V_max_/K_m_ and listed in Table 1. As a substrate for the CYP isozymes, the CL_int_ per nmoL of P450 for CYP2C9 is highest for both α-mangostin and gartanin. Both α-mangostin and gartanin had the inhibitory properties against 1A2, 2C9, 2D6 and 3A4. The *K*_*m*_ of α-mangostin were predicted to be 6.740 and 0.556 μM for CYP1A2 and CYP2C9, respectively. The possible atomic sites for metabolism were illustrated in Figs. 8. The scores range from 0 to 1 with higher scores indicating a greater propensity of being a metabolic site. The possible chemical structures of phase I oxidation for α-mangostin were proposed in Fig. 9. By the way, the metabolite of gartanin was not found to be metabolized in phase I metabolism in LC/MS/MS identification although the prediction from the program was identified.

**Table 1:** The predicted *K*_*m*_ (*K*_*i*_) and *V*_max_ values in ADMET Predictor™ for gartanin and α-mangostin

CYP	Gartanin					α-mangostin				
	Inhibition	Substrate	*K*_*m*_ (μM)	*V*_*max*_ (nmol/min/nmol P450)	CL_int_^1)^ mL/min/nmol P450)	Inhibition	Substrate	*K*_*m*_ (μM)	*V*_*max*_ (nmol/min/nmol P450)	CL_int_^1)^ mL/min/nmol P450
1A2	✓	✓	34.300	0.397	0.0115	✓	✓	6.740	0.171	0.0254
2C9	✓	✓	0.648	0.083	0.128	✓	✓	0.556	0.033	0.0594
2C19	✕	✓	90.200	0.013	0.00014	✕	✓	41.400	0.014	0.00039
2D6	✓	✕	29.700	109.000	3.67	✓	✕	23.600	103.000	4.364
1) CL_int_ parameters were calculated using Vmax/Km.										
CYP		Gartanin				α-mangostin				
		Inhibition		Substrate	*K*_*i*_ (μM)	Inhibition		Substrate		*K*_*i*_ (μM)
3A4_mid		✓		✓	19.951	✓		✓		21.913
3A4_tes		✓		✓	4.274	✓		✓		2.793

*mid* midazolam; *tes* testosterone

## Discussion

In the present study, the possible involvement of the active transport system of two compounds (gartanin and α-mangostin) was demonstrated. The bi-directional studies were conducted across the HEKn cells. Mannitol was used as the marker compound to compare and clarify the permeation of the test compounds.

The result of in silico method showed that the P_app_ from the experiments, P_app, A-B_ of gartanin (9 μM) could not be detected and P_app, A-B_ of α-mangostin was approximately lower than predicted 3-fold. The two compounds have more ability that can permeate through the intestinal better than skin when using each compound alone. It is possible pathway that α-mangostin may act as a co-effector to improve the bioavailability of gartanin [19]. Then, the ADMET Predictor™ provided a human CYP450 enzyme kinetic models including *K*_*m*_ and *V*_*max*_ models for five important recombinant CYP isozymes which were 1A2, 2C9, 2C19, 2D6, and 3A4. The gartanin and α-mangostin were predicted to be the substrate of CYP1A2, 2C9, 2C19 and 3A4 and had the inhibitory properties against 1A2, 2C9, 2D6 and 3A4. *K*_*m*_ of α-mangostin on CYP1A2 was higher than a CYP2C9. Our results of the prediction were similar to previously studies by Foti et al. [20], which they reported that α-mangostin was metabolized primarily by CYP1A2 and was potent inhibited the CYP2C family of enzymes.

Moreover, based on the investigation of metabolites by LC-MS/MS system, mangostin might be metabolized via phase I metabolism (tri-oxidation reaction) which correlated to the results from the prediction. This might be due to the appropriate structure of mangostin that eases the oxidation. However, gartanin was found to be metabolized via phase II sulfation by LC-MS/MS identification, the results of which contradicted those from the in silico prediction. Although, based on its structure, gartanin could probably be metabolized via phase I oxidation, the level of metabolites might be lower than the detection limit of LC-MS/MS.

## Conclusion

Due to the increase in using Garciniam angostana L. as a complimentary medicine and cosmetics in dermatological products. Skin is the barrier for the penetration of the xanthones active compounds including α-mangostin and gartanin in the extract. The comprehension of permeability and skin first pass metabolism can help determine the bioavailability of these compounds. The application of in silico modelling and simulation within drug development is rapidly increasing in the R&D sector of the pharmaceutical industry and it is increasing possibility of using them in natural product research. Thus, LC-MC/MS and ADMET predictor™ were used and compared for prediction of possibility of cutaneous metabolism of these two compounds. The investigation of the metabolites by a LC-MS/MS system found that α-mangostin might be metabolized via phase I metabolism (tri-oxidation reaction) which correlated to the result from the prediction that from the program. For gartanin, it was metabolized via phase II metabolism in sulfation process. Although, many computer modeling methods of metabolic pathways have already been developed, they are not perfect. Similar to our findings, the in silico prediction and the in vitro cell culture study may not demonstrate perfect matching. We need more investigational efforts which are continuously developed in our laboratory. More efficient and reliable in silico methods are essential to motivate more studies in the field in the near future and they can help reduce the cost for complimentary medicine development.

## Abbreviations

BPE: Bovine pituitary extract
cells/cm^2^: Cell per square centimeter
COS: Cells-on-sheet
cps: Count Per Second
Da: Dalton
DMSO: Dimethyl sulfoxide
HEKn: Human epidermal keratinocyte (neonatal)
HKGS: Human Keratinocyte Growth Supplement
HPLC: High Performance Liquid Chromatography
K_m_: Michaelis-Menten constant
LC-MS/MS: Liquid Chromatograph-Mass Spectrometer
LDL: Low-density lipoprotein
MDCK: Madin-Darby Canine Kidney
ml: Milliliter
mM: Millimolar
MW: Molecular weight
ng/ml: Nanogram per milliliter
nm/sec: Nanometer per second
P_app_: Permeability coefficient
P_app, A-B_: Permeability coefficient from apical side to Basolateral side
V_max_: Maximum metabolic capacity
% v/v: Percentage of volume by volume
μg/ml: Microgram per milliliter
μm: Micrometer
